# Measurement delay associated with the Guardian® RT continuous glucose monitoring system

**DOI:** 10.1111/j.1464-5491.2009.02887.x

**Published:** 2010-01

**Authors:** C Wei, D J Lunn, C L Acerini, J M Allen, A M Larsen, M E Wilinska, D B Dunger, R Hovorka

**Affiliations:** * MRC Biostatistics Unit, Institute of Public Health, Department of Paediatrics, University of CambridgeCambridge, UK; † MRC Biostatistics Unit, Institute of Public Health, Institute of Metabolic Science, University of CambridgeCambridge, UK

**Keywords:** calibration, continuous glucose monitoring, Guardian® RT, minimal model

## Abstract

**Aims:**

Using compartment modelling, we assessed the time delay between blood glucose and sensor glucose measured by the Guardian® RT continuous glucose monitoring system in young subjects with Type 1 diabetes (T1D).

**Methods:**

Twelve children and adolescents with T1D treated by continuous subcutaneous insulin infusion (male/female 7/5; age 13.1 ± 4.2 years; body mass index 21.9 ± 4.3 kg/m^2^; mean ± sd) were studied over 19 h in a Clinical Research Facility. Guardian® RT was calibrated every 6 h and sensor glucose measured every 5 min. Reference blood glucose was measured every 15 min using a YSI 2300 STAT Plus Analyser. A population compartment model of sensor glucose–blood glucose kinetics was adopted to estimate the time delay, the calibration scale and the calibration shift.

**Results:**

The population median of the time delay was 15.8 (interquartile range 15.2, 16.5) min, which was corroborated by correlation analysis between blood glucose and 15-min delayed sensor glucose. The delay has a relatively low intersubject variability, with 95% of individuals predicted to have delays between 10.4 and 24.3 min. Population medians (interquartile range) for the scale and shift are 0.800 (0.777, 0.823) (unitless) and 1.66 (1.47, 1.84) mmol/l, respectively.

**Conclusions:**

In young subjects with T1D, the total time delay associated with the Guardian® RT system was approximately 15 min. This is twice that expected on physiological grounds, suggesting a 5- to 10-min delay because of data processing. Delays above 25 min are rarely to be observed.

## Introduction

In the last two decades, numerous approaches to minimally invasive continuous glucose monitoring (CGM) have been proposed and at least four devices have become commercially available [1–4]. A clinical benefit of CGM has been suggested [5,6]. Most recently, it has been demonstrated that CGM improves glycaemic control in adults with Type 1 diabetes (T1D), although some barriers to effectiveness of CGM in children and adolescents with T1D remain [7].

Most CGM devices utilize a biosensor embedded subcutaneously to measure interstitial glucose [8]. It is generally accepted that a concentration gradient exists between interstitial glucose and blood glucose with a range between 20 and 110% of blood glucose [9,10], although a 60% gradient is most likely [11]. CGM devices correct for this gradient through calibration based on self-monitored capillary glucose measurements. An impediment to real-time accurate CGM tracing is the existence of a physiological delay between blood glucose and interstitial glucose which has been estimated to be approximately 5–10 min [12], although a wider range may be possible [13,14]. In addition to the physiological delay, data processing and filtering [15,16] may result in a ‘technological’ delay. The combined (total) delay is then observed by CGM users.

At present, these combined delays are not known or are poorly understood for commercial CGM devices, but their characterization may be important in determining the clinical utility of CGM devices and facilitating more informative user training. In the present study, we employ a population-based compartment modelling approach [17–19,31] to determine the time delay associated with the Guardian® RT system (Medtronic MiniMed, Northridge, CA, USA) [20] in young subjects with T1D and to complement existing knowledge about numerical and clinical accuracy of this CGM device [21–24].

## Subjects and methods

### Subjects and study protocol

Twelve children and adolescents with T1D treated by continuous subcutaneous insulin infusion [male/female 7/5; age 13.1 ± 4.2 years; body mass index (BMI) 21.9 ± 4.3 kg/m^2^; glycated haemoglobin (HbA_1c_) 8.7 ± 2.0%; duration of diabetes 7.0 ± 4.5 years; duration on pump 1.9 ± 1.1 years; total daily insulin 0.89 ± 0.27 U/kg/day; mean ± sd] participated in a clinical research study conducted at the Wellcome Trust Clinical Research Facility, Addenbrooke’s Hospital, University of Cambridge, UK. Participants and, as appropriate, their carers gave informed consent/assent. The study was approved by the Ethics Committee.

At least 24 h prior to the study, a glucose sensor was inserted and, following a run-in period and calibration as suggested by the manufacturer, the Guardian® RT CGM system measured sensor glucose every 5 min. On arrival at the Clinical Research Facility at 16.00 h, a cannula was inserted in a vein of one arm and kept patent with sodium chloride 0.9%. Blood samples were taken every 15 min from 17.00 h until 12.00 h the following day. Blood was collected into a 1.3-ml tube containing sodium fluoride. The reference blood glucose concentrations were measured using a YSI 2300 STAT Plus Analyser (YSI Life Sciences, Yellow Springs, OH, USA). Two meals were eaten at 18.00 h and 08.00 h the following morning to maintain a normal carbohydrate intake. Prandial insulin boluses were given with the meal. The Guardian® RT was calibrated at approximately 17.00 h and then every 6 h using blood glucose measured on the YSI.

### Interstitial glucose kinetics

A compartmental model [25,26] was used to describe the relationship between blood glucose and interstitial glucose. The model is expressed by a linear differential equation


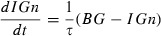
(1)

where *IGn* (mmol/l) is normalized interstitial glucose equalling blood glucose at steady state; the time constant *τ* (min) represents the delay between blood glucose and normalized interstitial glucose.

The intended clinical use of Guardian® RT is to approximate blood glucose. The calibration procedure residing on Guardian® RT aims to eliminate the blood-to-interstitial glucose gradient known to be approximately 60% at steady state [11], to mitigate the delay and to provide overall accurate glucose measurements.

### Calibration and measurement error

*IGn* is obtained by numerically solving Eqn (1) with *τ* as an unknown parameter to be estimated and *BG* given by linearly interpolating between the observed values. The unknown time delay *τ* is allowed to vary between individuals but is assumed constant for any given individual. To account for calibration, we assume that sensor glucose is linearly related to *IGn*, with intercept and gradient terms referred to throughout as the calibration shift and scale factor, respectively. These are unknown parameters, which are allowed in our model to differ between both individuals and calibration periods. Any residual discrepancies between the observed and modelled sensor glucose values are then assumed to be normally distributed with mean zero and with an unknown variance (to be estimated) that also varies between both individuals and calibration periods.

### Data analysis

All of the aforementioned unknown parameters are assumed to arise from ‘population distributions’ that characterize the mean and variability of the individual- and calibration-period-specific values. We use a population-based parameter estimation method in which all of the individual-, calibration-period- and population-level parameters are estimated simultaneously, which allows for more efficient usage of the information contained in the data set and typically leads to more reliable inferences. More details about the data analysis are provided in the Supporting Information (Appendix S1).

## Results

### Glucose profiles, glucose kinetics, and model fit

The reference blood glucose levels were 8.0 ± 2.8 mmol/l (mean ± sd) ranging from 3.0 to 18.0 mmol/l. Overall, 7% of the time blood glucose was ≤ 3.9 mmol/l, 51% of time blood glucose was between 3.9 and 8.0 mmol/l and 42% of the time blood glucose was ≥ 8.0 mmol/l.

**FIGURE 1:**
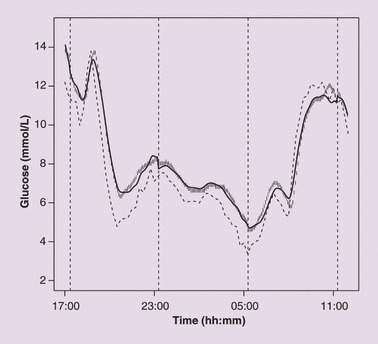
Blood glucose (dashed line), sensor glucose reported by Guardian® RT (vertical bar line) and model fit (solid line) in a young subject with Type 1 diabetes (individual number 2). Four vertical dashed lines indicate the times at which calibration was carried out, starting at approximately 17.00 h and then every 6 h. The estimated time delay between blood and sensor glucose *τ* for this subject is 15.2 min. Estimates for the calibration shift and scale factor in each of the five calibration periods are: 3.48, 1.96, 1.73, 1.74, −0.347 mmol/l (shift); and 0.784, 0.882, 0.826, 0.814, 1.04 (scale; unitless).

Differences between blood and sensor glucose were identified as demonstrated in Fig. 1, which shows blood and sensor glucose profiles for individual number 2. Mean sensor glucose is higher than blood glucose: the mean ± sd difference between sensor and blood glucose, in all individuals, is 0.24 ± 1.3 mmol/l. This results primarily from a positive calibration shift and a calibration scale factor below unity as reported for the majority of calibration periods. Sensor glucose generally lags behind blood glucose but, importantly, the degree of lag appears to change over time. However, the apparent lag reflects the extent of shifting and scaling as a result of calibration as well as the actual (kinetic) lag. Although our model is somewhat complicated, it aims to break down this apparent lag into its component parts. Hence, we may disentangle the effects of calibration and kinetic delay and better characterize the observed differences between blood and sensor glucose than would be possible through estimation of some ‘average’ apparent lag, via, for example, minimization of the differences between blood glucose and time-shifted sensor glucose [8,24]. Aside from providing a better understanding of the underlying processes, this represents an important step towards facilitating realistic prediction of blood glucose-sensor glucose profiles, which can be used, for example, to test glucose control algorithms [27]. Note that the good fit obtained by assuming a constant time lag *τ*, as shown by the solid line in Fig. 1, demonstrates little or no evidence of a time-varying kinetic delay. The model fit for this individual is fairly typical.

### Population analysis

Individual and population estimates for the time delay between blood and sensor glucose are given in Table 1, along with their interquartile ranges (IQR), which show the degree of precision.

**Table 1:** The median and interquartile range (reflecting accuracy of estimation) of the time delay *τ*

	*τ* (min)		
	
Subject number	Median	25% percentile	75% percentile
1	16.1	15.5	16.8
2	15.2	14.7	15.6
3	15.6	14.6	16.8
4	15.7	14.5	16.9
5	19.9	19.4	20.4
6	14.3	13.8	14.9
7	16.7	16.2	17.1
8	16.8	16.0	17.6
9	21.4	20.2	22.5
10	10.8	10.4	11.2
11	14.2	13.9	14.5
12	16.2	15.4	17.0
Population	15.8	15.2	16.5

Individual and population estimates are shown.

The individual delays range from 10.8 to 21.4 min. The population median delay is 15.8 min, with an IQR of (15.2, 16.5) min. To corroborate our results, we calculated the correlation coefficient between blood glucose and sensor glucose using a pure time lag of 0, 5, 10, 15 and 20 min. This was carried out by delaying sensor glucose in 5-min intervals. This is possible as sensor glucose is reported by Guardian® RT with a 5-min resolution. With a value of 0.937, the correlation coefficient peaks at 15-min delay, supporting our model-based estimate of the time delay.

The variability in the time delay is most usefully expressed by a predictive distribution for a new individual’s time delay. This is readily obtained from our analysis. Expressed as 2.5, 25, 50, 75 and 97.5% percentiles, the delay is 10.4, 13.9, 15.8, 18.1 and 24.3 min, respectively. Hence, with probability 0.95 a new individual’s time delay will lie between 10.4 and 24.3 min.

The population-based analysis also provides population and individual estimates for the calibration scale factor and shift, and the standard deviation *σ* of the residual errors. Population medians (IQR) for the scale and shift are 0.800 (0.777, 0.823) (unitless) and 1.66 (1.47, 1.84) mmol/l, respectively. There exists substantial variability between calibration periods, again best expressed through predictive distributions. Ninety-five per cent predictive ranges (PR95) for scale and shift are (0.437, 1.46) (unitless) and (–2.07, 5.41) mmol/l, respectively. Further statistical analyses suggest that the scale and shift are not characteristics of an individual; that is, none of the variability among their values appears to be attributable to differences between patients. The population median (IQR) residual standard deviation *σ* is 0.250 (0.236, 0.265) mmol/l, whereas the PR95 for new *σ*s is (0.0736, 0.842) mmol/l.

## Discussion

Our results indicate that, in young subjects with T1D, the delay between blood glucose and sensor glucose reported by the Guardian® RT CGM system is approximately 16 min and this result is supported by the correlation analysis. The highest correlation with blood glucose is observed when sensor glucose is delayed by 15 min. The delay has a relatively low intersubject variability, with 95% of individuals predicted to have delays between 10.4 and 24.3 min.

The delay comprises two components. The physiological delay reflects the diffusion of glucose from the plasma to the interstitial fluid and the data processing component reflects filtering the measurement noise and smoothing the sensor signal by software residing on the Guardian® RT. The consensus is that the physiological delay in adults is approximately 5–10 min [25], but may be shorter in young subjects with T1D. This suggests that the error filtering and data smoothing component of the delay is 5–10 min or longer and therefore of at least the same magnitude as the physiological delay. Therefore, the apparent delay cannot be deduced simply from the physiological delay, but is device-dependent, as different manufactures will use different data processing techniques.

Considering the population median scale factor at 0.800 (unitless) and the median shift at 1.66 mmol/l, the Guardian® RT calibration procedure will tend to match blood and sensor glucose at approximately 8.3 mmol/l. This is in agreement with a multi-centre study assessing numerical accuracy of Guardian® RT for children with T1D [21]. Below this threshold, Guardian® RT is likely to overestimate and above the threshold to underestimate blood glucose. However, high variability between calibration periods exists and this appears not to be subject specific. In our study, we used the highly accurate YSI for calibration. The use of less accurate glucose meters will further increase this variability.

Recently, a delay of 21 ± 5 min has been reported for the Guardian® RT in adults with T1D [24]. This appears to be longer than that reported in our results. However, the longer delay was estimated using an inappropriate methodology, i.e. the estimation involved ‘holding the YSI curve constant and then time shifting the CGM curve to minimize the error between YSI and CGM’ [24]. This visually guided procedure has three main limitations: (i) it is possibly subjective; (ii) it neglects the effects of scaling and shifting because of calibration on the differences between blood and sensor glucose: ideally these would be removed before (or simultaneously with) time-shifting, but their magnitudes are unknown; and (iii) it does not quantify the degree of uncertainty associated with the resulting delay estimate, which can lead to an unreliable characterization of the distribution of delays throughout the population of interest, as discussed below. We were able to separate the effects of calibration and delay, determining the scale, shift and underlying lag using a statistically rigorous approach without the need for subjective decision making. The resulting parameter estimates better characterize the observed differences between blood and sensor glucose.

The additional strength of our approach is that we estimate individual and population values in a one-stage process known as a hierarchical analysis [31]. This typically yields more reliable inferences than the alternative two-stage approach, where each subject is analysed independently and the population mean and variability are derived from the individual estimates. Any uncertain individual estimates, which may be somewhat unrepresentative of the underlying ‘true’ values, can unduly influence estimation of the mean and variability as the uncertainty is not taken into account, i.e. individual estimates contribute in the same way irrespective of their uncertainty. Typically, the two-stage estimate of the population variance is inflated when there is substantial uncertainty. By contrast, the one-stage approach allows ‘borrowing of strength’ across individuals, whereby well-determined individual estimates contribute more to the estimation of the population characteristics, and the information provided about these characteristics, in turn, helps strengthen the less well-determined individual estimates. This has the effect of increasing robustness of the population estimates as well as reining in the more unreliable individual estimates. In the context of the present study, subjects with a relatively constant or slowly changing glucose profile provide an uncertain estimate of the delay. Such uncertainty may explain the high variability and large delays obtained previously from two-stage analyses [13,14].

The expected differences between sensor and blood glucose depend on the rate of change of the glucose concentration. When glucose is changing rapidly, differences will increase. This may be perceived as an increase of the ‘apparent’ delay although the ‘kinetic’ delay remains unchanged. Our estimate of the kinetic delay allows the differences between sensor and blood glucose to be assessed. As an illustration, consider three different rates of glucose increase: 0.025, 0.05 and 0.1 mmol/l/min (the latter two rates are used by the Guardian® RT system to denote rapid or very rapid glucose change). The population median delay (15.8 min) then corresponds to differences between sensor glucose and blood glucose of 0.395, 0.790 and 1.58 mmol/l, respectively. Note that the difference increases in proportion to the rate of change of glucose even although the underlying delay is constant. Corresponding differences for the 2.5 and 97.5% percentiles (10.4 min, 24.3 min) of the predictive distribution for delay are (0.260, 0.608), (0.52, 1.22) and (1.04, 2.43) mmol/l, respectively. In practice, the differences will be inflated by the calibration error, which is expected to be relatively constant over a calibration period.

Using the model we developed, we obtained an excellent fit to sensor glucose in most individuals. However, the residual error was occasionally consistently positive or consistently negative, indicating the presence of unmodelled processes of uncertain origin. Our model could be expanded by assuming an autoregressive process for the residual errors instead of assuming them to be independent, or other approaches could be used to handle autocorrelated residuals [28].

The main objective of our study was to investigate the delay between blood and sensor glucose, which, in principle, should be independent from the way blood glucose is measured. Compared with a standard glucometer, the YSI provides more accurate measurements and thus the assessment of the delay should be highly accurate. However, additional errors associated with the use of capillary blood glucose meters may cause higher discrepancies to be observed in daily practice.

Our study complements the traditional assessment of clinical and numerical accuracy utilizing the error grid analysis, the assessment of absolute and relative absolute deviations and the correlation analysis [8,22,29,30]. In particular, our analysis provides useful information about the expected delay between blood and sensor glucose and the source and extent of the calibration error. The results were obtained in a heterogeneous population of young subjects with T1D, e.g. age ranged from 5 to 18 years, HbA_1c_ from 6.5 to 13.3% and the total daily dose as a marker of insulin sensitivity from 0.50 to 1.29 U/kg/day.

In conclusion, we adopted a population-based modelling approach to describe the delay between blood and sensor glucose reported by Guardian® RT. Our results suggest a typical delay of 15.8 min, with 95% of individuals predicted to have delays in the range 10.4–24.3 min. The delay is double that expected on physiological grounds, suggesting 5- to 10-min delays as a result of the data processing software residing on Guardian® RT.

## Abbreviations

CGM: continuous glucose monitoring
HbA_1c_: glycated haemoglobin
IQR: interquartile range
PR95: 95% predictive range
T1D: Type 1 diabetes

## Supporting Information

**Appendix S1.** Statistical model and implementation.
