# Supplementary material for: Measurement delay associated with the Guardian® RT continuous glucose monitoring system
Source: Diabet Med. 2010 Jan;27(1):117–22. doi: 10.1111/j.1464-5491.2009.02887.x (PMC3427888; doi:10.1111/j.1464-5491.2009.02887.x)
Supplement: Supplementary file 1 [file dme0027-0117-SD1.doc]

## APPENDIX

Let *i* index individuals; we denote the set of unknown time delays by *τ_i_*, *i* = 1,…,12. We further denote the sets of calibration shifts, scale factors and residual standard deviations by *B_ij_*, *F_ij_* and *σ_ij_*, respectively, where *j* indexes calibration periods (*j* = 1,…,5). The *k*th measured sensor glucose concentration for individual *i*, *SG_ik_* (*k* = 1,…,~230), is related to the corresponding normalized IG (*IGn_ik_*) according to

*SG_ik_* = *F_ip_*[*_i_*,*_k_*]*IGn_ik_* + *B_ip_*[*_i_*,*_k_*] + *ε_ik_*,

where *p*[*i*,*k*] ∈ {1,2,3,4,5} denotes the calibration period to which the *k*th observation for individual *i* belongs, and *ε_ik_* is a residual error with zero mean and variance *σ_ip_*[*_i_*,*_k_*]^2^. Due to their inherent positivity, the 1/*τ*, *F* and *σ* parameters are assumed to arise from log-normal distributions with unknown mean and variance parameters (*m.* and *v.*) to be estimated, whereas the *B* parameters are assumed to arise from a normal distribution with an unknown mean *m_B_* and variance *v_B_*, also to be estimated. As the *B*s and *F*s represent intercepts and gradients, respectively, which may be correlated, we also model the inter-individual/calibration-period covariance *c_FB_* of *B* and log*F* via a bivariate normal distribution (BVN).

$$\begin{matrix} \log\left(1/\tau_{i}\right)\sim\mathrm{N}(m_{\tau},v_{\tau}), \log(\sigma_{ij})\sim\mathrm{N}(m_{\sigma},v_{\sigma}), \\ \left(\begin{matrix} \log(F_{ij}) \\ B_{ij} \end{matrix}\right)\sim\mathrm{BVN}\left(\left(\begin{matrix} m_{F} \\ m_{B} \end{matrix}\right), \left(\begin{matrix} v_{F} & c_{FB} \\ c_{FB} & v_{B} \end{matrix}\right)\right), \\ i=1,...,12, j=1,...,5, \end{matrix}$$

Note that the log-normality assumption is the reason for our reporting population medians as opposed to means.

We implement the model using the WinBUGS software [1], which provides Bayesian estimates of the posterior distribution of unknown parameters. The posterior distribution simply summarises the uncertainty regarding each parameter’s value that remains after analyzing the data [2]. We use this approach for the flexibility that it offers in terms of underlying modelling assumptions. We use a specialized interface *WBDiff* [3] for defining the model in terms of differential equations.

References

1. Lunn DJ, Thomas A, Best N, Spiegelhalter D. WinBUGS – a Bayesian modelling framework: concepts, structure, and extensibility. Stat Comput 2000; 10: 325–337.

2. Gelman A, Carlin JB, Stern HS, Rubin DB. Hierarchical models. In: Xxxxx X, eds. Bayesian Data Analysis. Second edn. London: Chapman & Hall, 2003. [AUTHOR: Cambridge has been added as a location for this publisher - please amend if not correct.]

3. Lunn DJ. WinBUGS Differential Interface –Worked Examples. Cambridge: Department of Epidemiology and Public Health, 2004.
